# Loss of Independence Among Racially and Ethnically Diverse Older Adults: An ICF-Based Analysis of NHATS

**DOI:** 10.3390/healthcare14172710

**Published:** 2026-08-25

**Authors:** Young-Shin Lee, Seon-Hi Shin, Alison Moore, Leticia Camacho, Juan A. Cruz, Hee-Jin Jun

**Affiliations:** 1 School of Nursing, San Diego State University, San Diego, CA 92182, USA; 2 NYU Department of Radiology, Grossman School of Medicine, New York, NY 10016, USA; shinpaulineny@gmail.com; 3 School of Medicine, University of California San Diego, La Jolla, CA 92093, USA; alm123@health.ucsd.edu; 4 School of Public Health, San Diego State University, San Diego, CA 92182, USA; licamacho@sdsu.edu (L.C.); jcruz0044@sdsu.edu (J.A.C.J.); hjun@sdsu.edu (H.-J.J.); 5 Institute for Behavior and Community Health, San Diego State University, San Diego, CA 92182, USA

**Keywords:** loss of independence, environment, social participation, physical performance, reduced inequalities, good health and well-being

## Abstract

**Highlights:**

**What are the main findings?**

- Physical performance is the strongest direct association with LoI, operationalized using VES-13 across NH White, NH Black, and Hispanic older adults.
- In contrast, the relative importance of social and environmental factors differed across groups: social participation restriction was particularly prominent among NH Black adults, whereas limited English proficiency was particularly important among Hispanic adults.

**What are the implications of the main findings?**

- Preventive healthcare policies must move beyond generic, illness-focused models toward culturally responsive interventions tailored to specific community vulnerabilities.
- The ICF framework provides a useful approach for identifying potential intervention targets, including social engagement and linguistic accessibility, that should be further evaluated in longitudinal research.

**Abstract:**

**Background:** Preventing mobility-related loss of independence (LoI) is a critical public health priority. While the drivers of LoI are complex, this study examines how personal, social, and environmental factors influence these outcomes to identify actionable preventive targets. We applied the International Classification of Functioning, Disability, and Health (ICF) framework to examine these pathways across racial and ethnic groups. **Methods:** Using 2023 National Health and Aging Trends Study (NHATS) data (N = 7106), we conducted path analyses among non-Hispanic (NH) White, NH Black, and Hispanic older adults. We assessed associations between health conditions, body function, physical performance, social participation, and environmental factors. **Results:** The ICF-based model explained 36.6–38.2% of LoI variance. While physical performance was the strongest direct predictor of independence across all groups, indirect pathways varied. Social participation restrictions are associated with higher LoI score among NH White and Black adults. For Hispanic adults, limited English proficiency functioned as a key environmental variable negatively associated with body function and physical performance. Additionally, shared living arrangements among NH Black participants were negatively associated with physical performance. By gender, only NH White females exhibited associations with better physical and social outcomes. **Conclusions:** Pathways to LoI are associated with group-specific structural factors. To effectively prevent functional decline, healthcare policies should move beyond generic services toward culturally responsive interventions. Prioritizing targeted lifestyle behaviors—specifically social engagement for NH Black populations and linguistic accessibility for Hispanic adults—may help reduce health inequities and proactively preserve independence.

## 1. Introduction

Disability in later life is a significant contributor to loss of independence (LoI), posing challenges both nationally and individually [1]. Currently, 61 million Americans live with at least one disability, including 43.9% of those aged 65 and older [2,3,4]. As individuals age, the prevalence of disability increases—from 24% at age 65 to 46% at age 75 and 75% at 85 and older [2,5,6]. Combined with rising life expectancy, this trend presents a substantial public health concern [1]. Mobility disability is the most frequent type, affecting approximately 27% of older adults in the U.S. [3,7]. This prevalence climbs sharply with advancing age, impacting up to 46% of individuals 75 and older and over 70% of those aged 90 and older [1,8]. The longer we live, the more likely we are to experience mobility disability due to a health condition or injury [9]. This is often heightened by reduced social and community engagement as we age. It is a primary contributor to LoI and disproportionately impacts those of advanced age, women, individuals below the poverty line, Latinx populations, and those living alone [1,3,6,7]. Women, in particular, experience more limitations in daily activities and are more likely than men to lose independence as they age [10,11]. Over the past decade, the average duration of dependency in women has increased from 10.4 to 10.9 years. Understanding racial and ethnic disparities in disability remains complex, as these differences may reflect socioeconomic, social, environmental, and health-related factors [12]. Non-Hispanic (N-H) Blacks and American Indian/Alaska Natives experience higher prevalence of various disabilities than other groups [3,12]. To date, there is still limited research on LoI disparities among Hispanics and Asian Americans.

Although functional decline and late-life disability are complex and multidimensional, they are partially preventable and modifiable [10,13]. Reflecting this shift, healthcare systems in developed countries have moved beyond illness-focused care to emphasize prevention and the promotion of functioning in older adults with chronic conditions [14,15]. To guide this broader approach, the World Health Organization’s International Classification of Functioning, Disability and Health (ICF) model provides a standardized, holistic framework for understanding disability and functioning, emphasizing a holistic approach [16,17,18]. This person–environment perspective makes the ICF particularly well suited to the study of racial and ethnic disparities in disability. Rather than locating disability solely within individual health conditions or impairments, the ICF conceptualizes functional outcomes as arising from the interplay among health conditions, body function, activity and participation, and contextual factors. Social and environmental circumstances, including language-related barriers and living arrangements, may differ across racial and ethnic groups and influence how health conditions and impairments are associated with functional outcomes. This framework therefore provides a theoretical basis for examining whether pathways to Loss of Independence differ across racial and ethnic groups rather than assuming a single, universal mechanism of functional decline [12,16]. The model integrates physical, psychological, social, and environmental factors as they influence individuals’ daily functioning [16,18]. Widely used in developed countries, the ICF has demonstrated efficacy in guiding comprehensive care plans and rehabilitation strategies that extend beyond traditional medical assessments [19,20].

In the U.S., national efforts to reduce disability have aligned with this framework through initiatives such as Healthy People 2010, which promotes a holistic view of well-being [1,17,21,22]. Federal agencies have also adopted standardized methods to assess disability, including self-reported measures across six functional domains [23,24]. A key initiative, the National Health and Aging Trends Study (NHATS), was launched to collect longitudinal data on a nationally representative sample of Medicare beneficiaries aged 65 and older. NHATS integrates core principles of the ICF model in its design [25]. While several recent studies have applied the ICF model using NHATS data [26,27,28,29], few have examined a comprehensive approach to understanding how older adults maintain independence within this framework. A more integrated application of the ICF is needed to fully capture the multifactorial nature of independence in late-life.

To address gaps in both research and preventive healthcare, this study applies the ICF model to data from the NHATS to identify predictors of loss of independence (LoI) among multiethnic older adults living independently in the U.S. Specifically, it aims to: (1) examine how impaired body function, physical performance, and restricted social participation contribute to LoI; and (2) assess whether these factors mediate the effects of gender, health condition, living arrangements, and English proficiency on LoI among non-Hispanic (N-H) White, NH Black, and Hispanic groups. Findings may inform the development of culturally responsive, function-focused interventions to support independence and reduce disparities in aging populations.

Based on the ICF framework, we hypothesized that: (1) impaired body function, lower physical performance, and restricted social participation would be directly associated a higher risk of LoI across all groups; and (2) these functional and social domains would account for indirect associations between personal factors (e.g., gender, health conditions) and environmental factors (e.g., living arrangements, English proficiency) on LoI, with the specific indirect pathways varying among NH White, NH Black, and Hispanic older adults.

## 2. Materials and Methods

**Figure 1:**
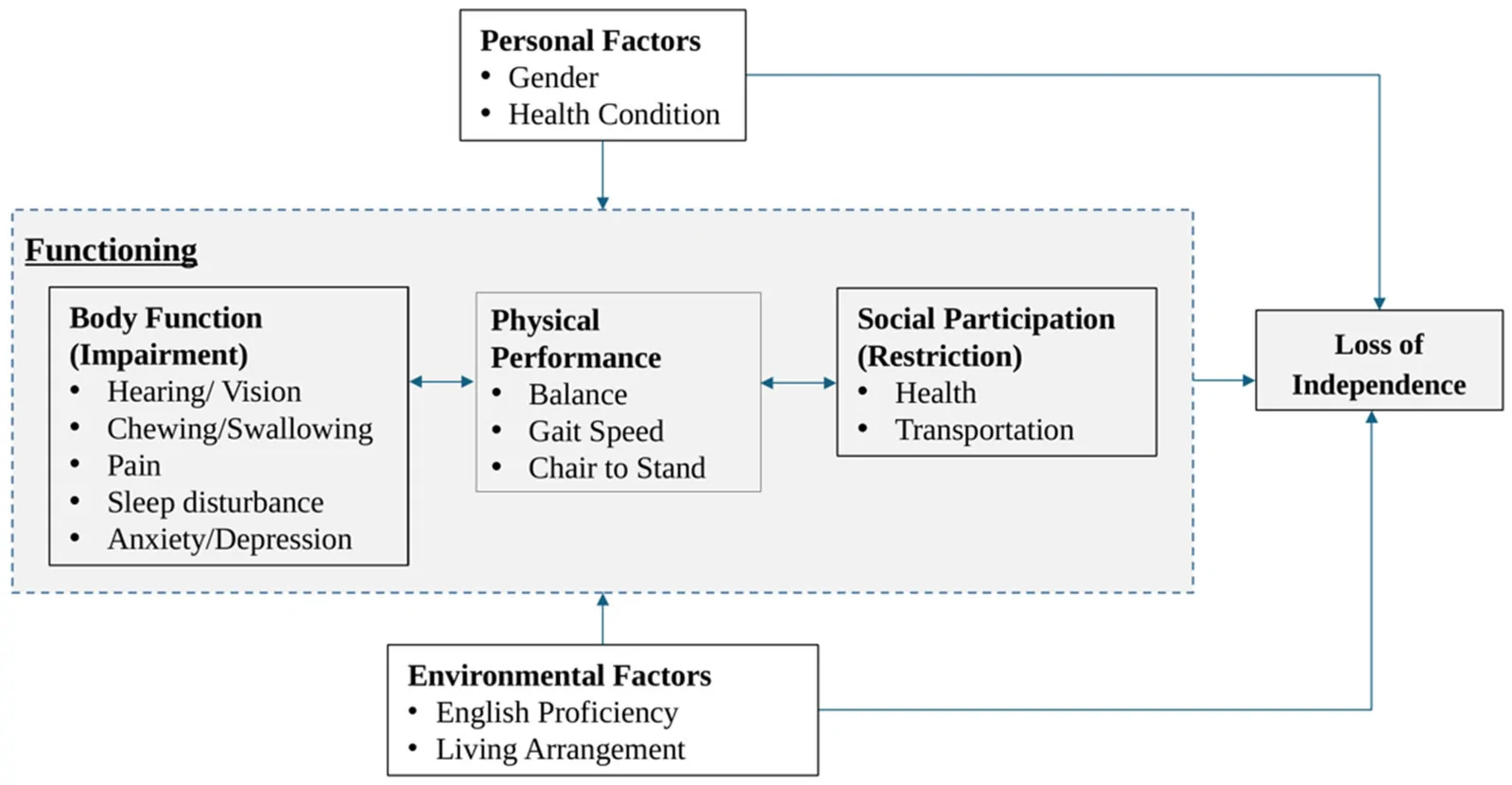
Theoretical Framework, adapted from the International Classification of Functioning, Disability and Health (ICF) Model.

**Study Design:** The proposed research employs a population-based cross-sectional design to investigate risk factors predicting the loss of independent life under the ICF theoretical framework. Figure 1 illustrates a comprehensive approach that considers health condition, body function and structure, physical performance, social participation, and environmental factors in accordance with the ICF model.

**Data and Sample Description:** This study utilized Round 13 (2023) Sample Person (SP) data files from the National Health and Aging Trends Study (NHATS), a longitudinal study initiated in 2011. NHATS employs a stratified, multistage sampling design based on geographic clusters across the United States to provide a nationally representative cohort of Medicare beneficiaries aged 65 and older [30]. Although participants were enrolled across multiple waves, our analytic sample was based on data collected in 2023. Participants were included if they responded on their own behalf; nursing home residents were excluded to ensure the focus remained on those living independently in community settings. **Sample:** The final analytic sample comprised 7106 community-dwelling older adults categorized into three racial and ethnic groups: Non-Hispanic (NH) White, NH Black, and Hispanic. To derive this sample from the original 8597 Round 13 participants, a total of 1491 cases (17.3%) were excluded: 485 individuals (5.6%) who did not meet the race/ethnicity eligibility criteria (identifying as groups other than NH White, NH Black, or Hispanic), and 1006 otherwise eligible participants (11.7%) with missing data on key model variables (574 missing outcome data on the VES-13 and 432 missing physical performance data on the SPPB). No further participants were excluded. All continuous variables were coded such that higher values indicate poorer status (e.g., impaired body function, lower social participation, poorer health conditions, and lower English proficiency), except for physical performance, where higher scores indicate better functioning. The same measurement procedures and coding rules were applied across all racial and ethnic groups.

**Outcome measure:** Loss of Independence (LoI) was operationalized using the Vulnerable Elders Survey-13 (VES-13), a function-based screening instrument developed to identify community-dwelling older adults at increased risk of future function decline or death. Because the VES-13 incorporates age, self-rated health, physical capacity, and difficulty performing independent functional daily activities, the total score (ranging from 0 to 10) was used as a continuous indicator of LoI-related functional vulnerability in the present path analyses. The 13-item questionnaire aligns with NHATS variables and assesses age, self-rated health, difficulty in physical capacity (6 items), and difficulty in physical performance (5 items). Points from these four components are summed to produce the total continuous score. Age was classified into three groups (young-, middle- and old-old), assigning a score of “0” for age < 75, “1” for 75–84, and “3” for 85 or older. Self-rated health offered five options: poor, fair, good, very good, or excellent. Those reporting poor or fair received 1 point, while all others received 0 points. Difficulties in physical capacity were assessed through six self-reported activities: kneeling, carrying, reaching, grasping, walking 3 blocks, and lifting a heavy object. Each reported difficulty received 1 point, and total scores were categorized as 0, 1, or 2 points (with 2 points representing difficulty in two or more activities marked as ‘yes’). Difficulties in physical performance were evaluated using five items related to independent functioning: shopping, handling finances, mobility, laundry, and showering. If participants reported difficulty in any activity, they were assigned 4 points. The VES-13 total score ranges from 0 to 10 with higher scores indicating an increased risk of health vulnerability. While a cut off score of 6 or higher has been used to denote high risk of functional vulnerability [31,32], the present study modeled LoI as a continuous outcome to preserve variability across the full range of scores rather than dichotomizing it. Previous validation studies conducted in the general community-dwelling population have reported VES-13 sensitivity ranging from 67% to 91% and specificity from 59% to 79% [33,34].

**Personal factors:** This domain includes demographics and selected health conditions. Demographic variables include gender (man and woman), race and ethnicity. Race/ethnicity was classified as non-Hispanic (NH) White, NH Black, Hispanic, or NH Other. The NH Other category combined participants identified as American Indian/Alaska Native, Asian American, Native Hawaiian/Pacific Islander, or another specified racial group. The analysis was limited to NH White, NH Black, and Hispanic participants; those classified as NH Other or unresolved or missing race/ethnicity were excluded. The health conditions domain includes self-rated health, number of chronic diseases, number of falls and fractures, and number of hospitalizations in the past 12 months. Chronic diseases are based on 10 medical diagnoses (heart attack, heart disease, high blood pressure, arthritis, osteoporosis, diabetes, lung disease, stroke, dementia/Alzheimer’s disease, cancer) [25]. Responses are binary (yes/no), with total scores categorized as light comorbidity (0–2 conditions) or severe comorbidity (3 or more conditions).

A history of falls, fractures and hospitalizations in the past year was recorded. Responses were binary (yes/no), summed into a 0–4 score, with higher values indicating increased health risk.

**Impaired body function:** This domain assessed limitations using NHATS data, focusing on sensory deficits, pain, fatigue, and mental health during the last month. Sensory function assessed difficulties with hearing, vision, and chewing/swallowing, including the use of assistive devices. Responses are ‘yes’ (1) or ‘no’ (0) with total scores ranging 0–3. Pain was measured using the question: ‘Is the person bothered by pain and does it limit their activities? (yes = 1, no = 0). Sleep quality was measured by participant reports of difficulty falling asleep (taking more than 30 min) most nights/every night (1) vs. less frequently (0). Depression and anxiety were assessed using the Patient Health Questionnaire (PHQ-4), a validated screening tool combining PHQ-2 (depressive symptoms) [35,36] and the Generalized Anxiety Disorder Screener (GAD-2) [23,30]. Responses range from 0 (not at all) to 3 (nearly every day) per item, with total scores ranging from 0–12. A cut-off score of ≥3 indicates probable depression or anxiety (yes = 1, no = 0).

**Physical performance** was assessed using the Short Physical Performance Battery (SPPB), a widely used measure of lower extremity function and mobility in older adults [37,38]. The SPPB includes standing balance, gait speed and the sit-to-stand test and is measured on a 12-point scale with higher numbers indicating better performance. Standing balance was assessed using three progressively different feet positions (side-by-side, semi-tandem, and tandem). Participants who maintained a full tandem stance for 10 s and completed other tasks scored 4 points [39]. Gait speed was measured as the time taken to complete a 3 m walk at a usual pace and then converted to the speed meter per second. Scores range from 0 to 4 with higher scores indicating better (faster) performance [40]. Sit-to-stand from a chair measured time taken to stand from a chair five times [41,42]. Each task is scored out of 4, with a total SPPB score from the three tests ranging 0 to 12. Lower scores indicate higher odds of mobility-related disability. This continuous 12-point scale was utilized in all subsequent path analyses.

**Social participation restriction** in NHATS was characterized by limitations on engaging in social activities in the past month due to health or transportation issues. Respondents reported whether they experience limited social engagement due to health issues of 7 types and/or transportation barriers in 4 activities. Responses were binary (yes/no). Each variable of the total 11 barriers was summed (0–11 points) but scores were capped at a maximum of 7 to account for statistical sparsity in the upper tail, thereby ensuring robust parameter estimation during path analysis.

**Environmental factors** included living arrangement (living alone, with spouse and/or other, or with only others) and English proficiency, which was assessed among participants in all racial and ethnic groups using two self-reported items on understanding and speaking English categorized as ‘very well/well’ and ‘not well.’

**Data Management and Statistical Analysis**

All variables utilizing the ICF framework underwent rigorous data verification to ensure accuracy and consistency. Collinearity and normality were assessed prior to analysis. To ensure valid and generalizable results, the analyses accounted for the NHATS complex sampling design, incorporating strata, clusters, and sampling weights. Specifically, all path analyses performed in Mplus (Ver. 8.11) explicitly incorporated these complex survey parameters for each racial/ethnic subpopulation to ensure unbiased standard error estimation. Subsequently, we conducted a comprehensive descriptive analysis on the entire sample, followed by separate analysis stratified by race/ethnicity to account for potential variations observed across different demographic groups. In descriptive statistics, we calculated means, standard deviations, and percentages as weighted and frequencies as the actual numbers of participants (unweighted) to understand how much data the estimates are based on.

To explain the comprehensive ICF model, we applied a path analysis by using the Mplus 8.11 program [43], which is widely used for modeling relationships among observed and latent variables, and for testing complex statistical models. Before conducting the path analysis, all preliminary analyses were conducted using SAS 9.4 [44]. We tested whether the associations between the predictor variables and LoI varied by racial/ethnic group using interaction terms between race/ethnicity and each predictor. Based on these preliminary interaction analyses, we proceeded with separate path analyses stratified by racial/ethnic groups. Prior to model estimation, we assessed the distributional properties of the continuous variables within each racial/ethnic group. Normality tests—Shapiro–Wilk [45], Kolmogorov–Smirnov [46], Cramer–von Mises [47], and Anderson–Darling [48]—indicated that the continuous variables were not normally distributed. As a result, we used the robust maximum likelihood estimator (MLR) in Mplus, which provides standard errors and model fit statistics that are robust to non-normality (Supplementary Table S1). To handle missing data, we conducted a complete case analysis within each group, excluding all cases with missing values on any predictor variables from the final dataset. To assess potential selection bias associated with complete-case analysis, we compared participants included in the analytic sample with otherwise eligible participants excluded because of missing VES-13 or SPPB data. Comparisons were based on weighted means and proportions for available demographic, health, functional, social, and environmental characteristics. Absolute standardized differences were calculated to evaluate the magnitude of group differences, with values of 0.10 or greater indicating potentially meaningful differences.

Multiple imputation was not part of the original analytic plan. It was not introduced retrospectively because missing VES-13 or SPPB data may reflect frailty, cognitive impairment, interview burden, or inability to complete the assessment that may not be fully captured by the observed variables, making the assumptions required for multiple imputations difficult to support. Participants outside the three pre-specified racial/ethnic groups were excluded by design and were not candidates for imputation. We therefore retained the complete-case approach and evaluated the potential for selection bias through the comparison described above. We also evaluated multicollinearity among predictors within each racial/ethnic group by examining bivariate correlations and calculating the variance inflation factor (VIF) for each predictor. All VIF values ranged from 1.0 to 1.7, well below the commonly used threshold of 5, indicating no evidence of problematic multicollinearity.

## 3. Results

### 3.1. Study Population

Table 1 summarizes the descriptive statistics of the study population. Demographics: The sample (n = 7106) was predominantly female (55.0%), aged 65–74 (52.2%), and identified as NH White (82.2%).

Loss of Independence (LoI): The overall mean score for LoI, measured by the Vulnerable Elders Survey (VES-13), was 3.87 (on a 0–10 scale), presenting moderate vulnerability. Statistically significant disparities were observed across ethnic groups (*p* < 0.001): Hispanic participants exhibited the highest risk (4.60), followed by NH Black (4.08) and NH White groups (3.77). Health conditions & Body (physical) function: Participants reported an average of 0.92 chronic conditions and 2.46 functional impairments across six domains. Notably, differences between racial and ethnic groups for these specific variables were not statistically significant (*p* = 0.19 and *p* = 0.51, respectively).

Physical performance: On a 12-point objective scale, the overall average score was 8.47 (SE = 0.09). NH White participants demonstrated significantly better performance (8.75, SE = 0.10) than both Hispanic (7.32, SE = 0.17) and NH Black participants (7.12, SE = 0.14; *p* < 0.001). This suggests that the determinants of LoI disparities may lie in functional execution rather than simple disease prevalence.

Social participation: Based on 11 health- and transportation- related restriction items, NH Black participants reported the highest average restriction score (0.82, SE = 0.05; *p* = 0.05), warranting inclusion in the path model as a theoretical mediator.

Among the 8112 participants who met the race/ethnicity eligibility criteria, 1006 were excluded because of missing VES-13 or SPPB data. Compared with participants included in the analytic sample, excluded participants had more health conditions (1.29 vs. 0.92; absolute SMD = 0.38), greater body function limitations (2.69 vs. 2.46; absolute SMD = 0.19), and greater social participation restriction (1.54 vs. 0.62; absolute SMD = 0.55). They were also less likely to live with a spouse (36.0% vs. 56.6%; absolute SMD = 0.42) or to be NH White (68.9% vs. 82.2%; absolute SMD = 0.31) and were more likely to be NH Black (15.3% vs. 9.0%; absolute SMD = 0.20) or Hispanic (15.7% vs. 8.8%; absolute SMD = 0.21).

### 3.2. Model Testing and Estimation

Preliminary interaction analyses showed significant race/ethnicity and physical performance interactions for NH Black (*p* = 0.001) and Hispanic participants (*p* < 0.001), relative to NH White participants. The race/ethnicity and social participation restriction interaction was significant for NH Black participants (*p* = 0.004), but not for Hispanic participants (*p* = 0.435). Interactions between race/ethnicity and impaired body function were not significant for NH Black (*p* = 0.306) or Hispanic participants (*p* = 0.868). These findings supported examination of the path models separately by racial/ethnic group.

**Figure 2:**
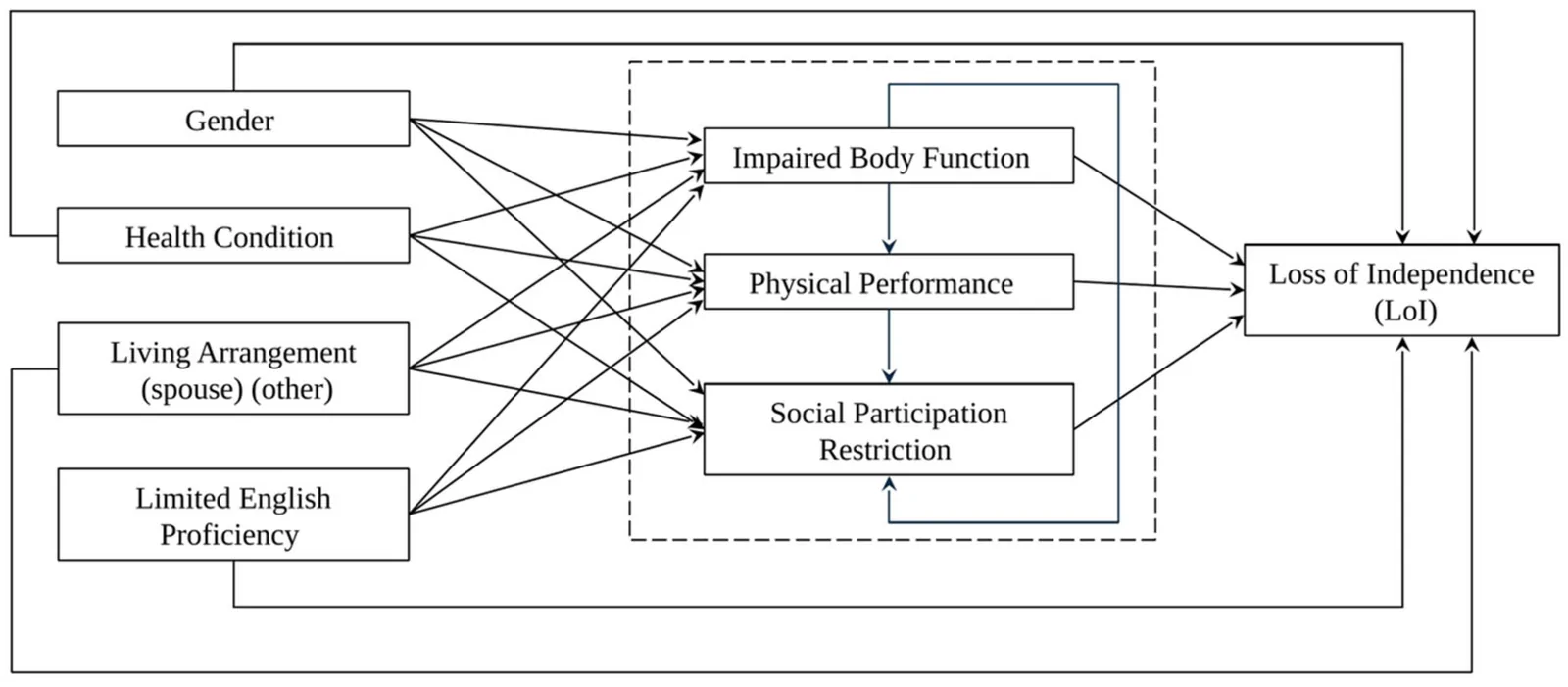
Hypothesized Statistical Framework.

Path analysis was conducted separately for each race/ethnic group using a four-step process to address the methodological complexities inherent in multicultural functional health. Step 1. Model Specification: The initial hypothesized model (Figure 2) defined directional relationships among ICF domains—including health conditions, body function, physical performance, and social participation—resulting in 33 free parameters for estimation. Step 2. Model Estimation and Fit Assessment: The model was initially treated as a saturated model. For each racial/ethnic group, we fitted several plausible models by varying predictors with direct paths to LoI while maintaining a consistent structure for indirect paths. We specified directional paths from body function to physical performance, body function to social participation, and physical performance to social participation. Step 3. Statistical Rigor: All models were estimated using the robust maximum likelihood estimator (MLR) to account for potential non-normality in the data. Model fit was evaluated using the Satorra–Bentler scaled chi-square difference test (TRd), the standardized root mean square residual (SRMR), the root mean square error of approximation (RMSEA), and the comparative fit index (CFI) [48,49,50,51]. Step 4. Final Model selection: Final model selection was guided by both theoretical relevance to the ICF framework and empirical fit criteria: (a) RMSEA < 0.06 and SRMR < 0.10, or (b) SRMR < 0.10 and CFI > 0.96 [48]. For all three ethnic/racial groups, the initial models with 33 estimated parameters were retained as they provided the optimal balance of theoretical coherence and statistical fit, explaining significant variance in LoI (NH White: 38.2%; NH Black: 37.0%; Hispanic: 36.6%), as detailed in the path coefficients below.

### 3.3. Path Analysis

Standardized coefficients and corresponding *p*-values are reported for the pathways discussed below. In the text, β denotes standardized direct-effect estimates, and γ denotes standardized indirect-effect estimates.

**Figure 3:**
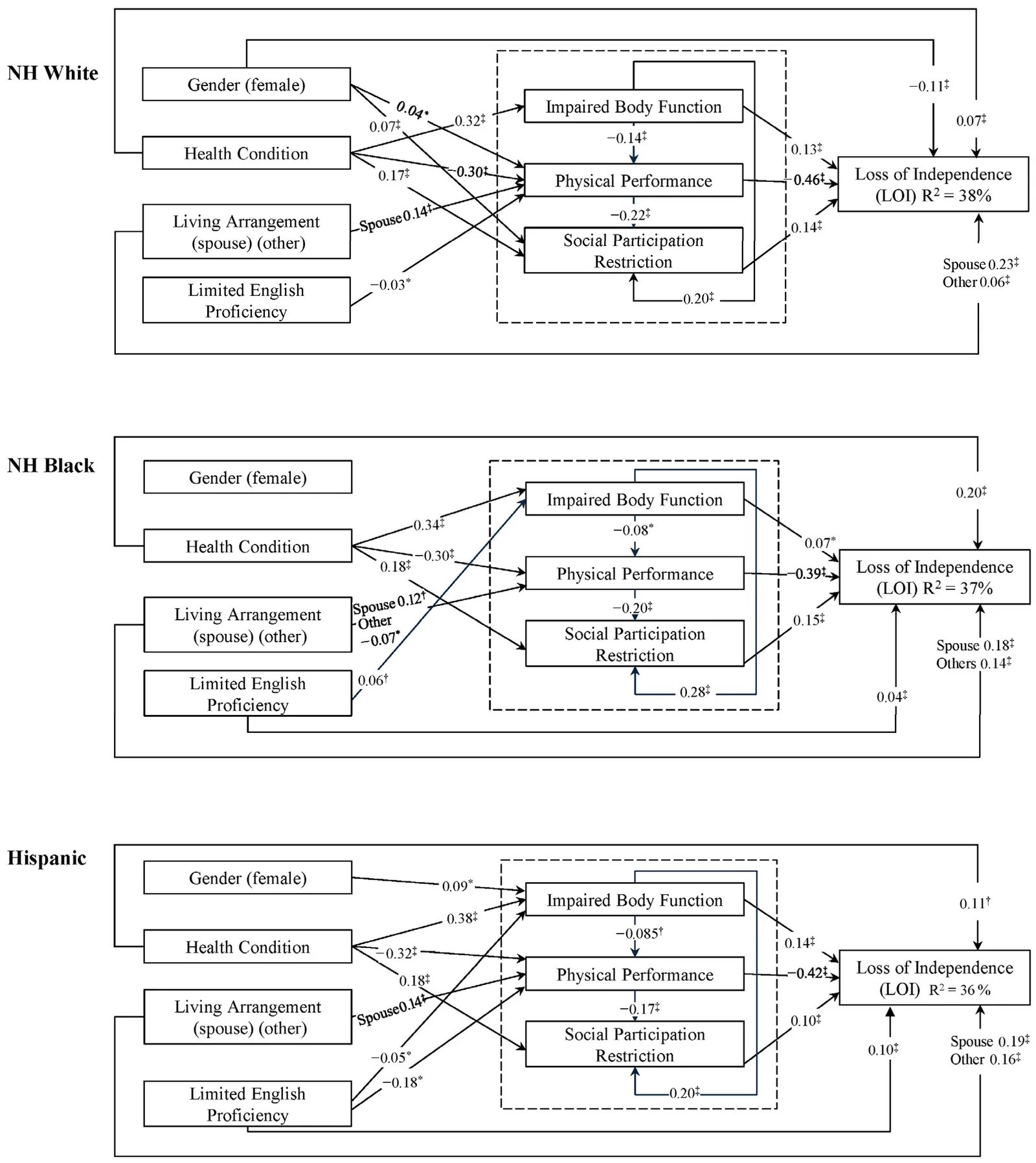
Path Models by Ethnic Groups. Note. Values are standardized path coefficients. * *p* < 0.05; *p* < 0.01; ‡ *p* < 0.001.

- ***Personal predictors of LoI***: Across the stratified models, LoI was significantly associated with gender, health condition, living arrangement and English proficiency (*p* < 0.01) (Table 2; Figure 3).

- ***Gender***: The impact of gender on functional independence varied significantly by race and ethnicity: Only NH White women exhibited a direct association with a lower risk of LoI compared to NH White men (β = −0.11, *p* < 0.001). This group also demonstrated significant indirect protective effects mediated through improved physical performance. NH Black women did not exhibit any significant direct or indirect effect on LoI (*p* > 0.05), suggesting that the gendered experience of disability may be moderated by other structural factors in this population. Hispanic women demonstrated an indirect effect on LoI mediated specifically through limited body function (γ = 0.012, *p* < 0.05).
- ***The Role of Health Conditions***: Health conditions were directly associated with a higher risk of LoI across all three groups. Health conditions also influenced LoI indirectly by increasing impaired body function, reducing physical performance, and increasing social participation restrictions (*p* < 0.001). The strongest direct effect was observed in the NH Black group (β = 0.202, *p* < 0.001), followed by the Hispanic and NH White groups (β = 0.110 and β = 0.074, *p* < 0.01, respectively). In the NH Black group, only health conditions had an indirect effect on LoI through body function limitations (β = 0.024, *p* < 0.05).
- ***Limitations in body function*** had a significant direct effect on LoI across all groups (β = 0.072~0.137, *p* < 0.001). An indirect association was also observed: poor health conditions are associated with body function limitations, which in turn are associated with a higher risk of LoI (γ = 0.024~0.052, *p* < 0.05). Among subgroup differences, only Hispanic women showed an indirect association with LoI through limitations in body function (β = 0.012, *p* < 0.05).
- ***Living arrangements***: Compared to those living alone, individuals living with spouse or with others (non-family members) had a significantly higher risk of LoI across all racial/ethnic groups (β = 0.06~0.23, *p* < 0.001). Additionally, those living with spouse exhibited an indirect effect on LoI through lower physical performance (γ = −0.05~−0.07, *p* < 0.01).
- ***Limited English proficiency*** had a direct effect on LoI in both NH Black and Hispanic groups (β = 0.04 and β = 0.10, *p* < 0.01, respectively).
- ***Physical performance*** as a Key Mediator. Consistent with the study hypothesis, physical performance emerged as a key structural correlate. Physical performance had a strong direct negative impact on LoI across all racial/ethnic groups (β = −0.39~−0.46, *p* < 0.001), indicating that better physical performance is the most robust predictor of maintained independence. Additionally, physical performance mediated the effects of poor health, body function limitations, and living with a spouse all groups (*p* < 0.01). Group-specific Indirect Pathways: Beyond the direct associations, the stratified model showed different patterns of significant indirect associations across racial and ethnic groups. Among NH White, female gender showed a significant indirect protective effect on LoI, mediated by superior physical performance (γ = −0.020, *p* < 0.05). In the NH Black groups, a unique social-environmental pathway emerged: Living with non-family others was indirectly associated with higher LoI through its negative impact on physical performance (γ = 0.027, *p* < 0.05). For the Hispanic group, limited English proficiency functioned as a significant structural barrier, increasing LoI risk indirectly through its negative association with physical performance (γ = −0.074, *p* < 0.05).
- ***Indirect Associations.*** As detailed in Table 2, path analysis identified indirect associations in which increased chronic conditions were associated with impaired body function, which was subsequently associated with reduced objective physical performance and, ultimately, higher LoI scores (γ = 0.010~0.021, *p* < 0.05).
- ***Social participation*** restriction had a significant direct positive effect on LoI across all racial/ethnic groups (β = 0.104~0.153, *p* < 0.001), indicating that greater restriction was consistently linked to a higher risk of losing independence. Health conditions had a significant indirect effect on LoI through social participation restriction across all groups (γ = 0.018~0.027, *p* < 0.001). This pattern indicated that poorer health was associated with greater social participation restriction, which in turn was associated with higher LoI score. Among demographic variables, only female gender among the NH White participants showed a significant indirect association with LoI through social participation restriction (γ = 0.009, *p* < 0.001). However, living arrangement (with spouse or others) and English proficiency did not show significant indirect effects through social participation pathway (*p* > 0.05) (Table 2).
- ***Total Variance Explained and Factor Contributions.*** The final ICF-based models explained 38.2% of the variance in LoI among NH White participants, 37.0% among NH Black participants, and 36.6% among Hispanic participants. The models also explained 20.7–23.8% of the variance in social participation restriction, 14.2–21.0% of the variance in physical performance, and 11.0–16.8% of the variance in impaired body function. The NH Black model explained the greatest proportion of variance in social participation restriction (23.8%), whereas the Hispanic model explained the greatest proportions of variance in physical performance (21.0%) and impaired body function (16.8%).

To understand the relative importance of these predictors, we examined the contribution of each factor to the explained variance (Table 2): Social participation restriction in all three ethnic groups accounted for the largest share of the explained variance (ranging from 20.7% to 23.8%). NH black group showed the highest contribution of social participation restriction to LoI (23.8%).

Physical performance accounted for 14.2%~21% of variance, followed by body function (11% to 16.8%, *p* < 0.001). Notably, the Hispanic group exhibited the greatest contributions from both physical performance (21%) and body function (16.8%) compared to the other groups, reinforcing the finding that physical determinants are particularly noticeable for this population.

## 4. Discussion

### 4.1. Theoretical Utility of the ICF Framework

Our findings support the applicability of the International Classification of Functioning, Disability, and Health (ICF) framework for examining Loss of Independence (LoI) across racially and ethnically diverse older adults. The models accounted for 36.6% to 38.2% of the variance in LoI across groups. This substantial explanatory power demonstrates that integrating health conditions, body function, physical performance, social participation, and contextual factors provides meaningful explanatory value beyond a purely medical lens [18,20,52]. By incorporating personal factors and environmental contexts, the ICF captures the dynamic nature of aging in a multicultural society, aligning with the core objective of the NHATS to provide a comprehensive view of late-life vulnerability [53]. While these models capture core functional and environmental pathways, remaining unexplained variance likely points to unmeasured structural and socioeconomic factors—such as income, built environment, and healthcare access—that warrant exploration in future datasets.

### 4.2. Racial and Ethnic Differences in LoI Risk

This study identified significant racial/ethnic disparities in LoI scores: Hispanic participants exhibited the highest vulnerability, followed by the NH Black and NH White participants. While some epidemiological literature reports the highest overall disability prevalence among NH Black older adults [3,12], our observed contrast likely stems from study design and sample composition.

Specifically, our analysis focused on community-dwelling individuals who responded on their own behalf and were assessed for risk of dependency in daily living using the continuous VES-13 score, which may reflect a healthy survivor effect within this independent, community-based cohort. More broadly, this variation may underscore that ethnic disparities in functional decline are highly context-dependent.

### 4.3. Universal vs. Group-Specific Predictors

Across all three racial and ethnic groups, while physical performance showed the strongest direct association across groups, the relative importance of the indirect pathways varied across groups. Specifically, the substantial variance accounted for by social participation restriction underscores that social and environmental “accelerants” are just as critical in determining functional trajectories, a pattern particularly prominent among NH Black participants. These findings are consistent with previous research showing that greater social engagement is associated with a lower risk of functional decline [54,55].

**NH White group**: A unique gendered pathway emerged where NH White women showed direct and indirect protective effects against LoI through enhanced physical performance and social participation.

This finding suggests that women in this cohort may exhibit different patterns of social engagement or health-seeking behaviors than their male counterparts, aligning with studies that highlight gender-based differences in late-life independence [56,57]. Conversely, living with a spouse was strongly associated with an increased risk of LoI (β = 0.23). Notably, this result contrasts with research reporting greater ADL independence among women living with spouses [58]. Given the cross-sectional design, this observational link likely reflects care-seeking transitions—where older adults experiencing functional decline remain with or transition to living with a spouse for support—rather than a causal dependency effect.

**NH Black group**: While gender showed no significant direct or indirect influence on LoI, social and environmental factors were paramount. Living with non-family members was uniquely associated with higher LoI through its negative relationship with physical performance. This pathway highlights complex trade-offs in multicultural households, where shared living arrangements may be shaped by economic or caregiving necessity, while simultaneously serving as markers of underlying physical vulnerability. Furthermore, the contrast between our findings and broader epidemiological reports of higher disability prevalence among NH Black older adults likely reflects our sample inclusion criteria. By focusing strictly on community-dwelling individuals who responded on their own behalf and excluding institutionalized populations, our sample may reflect a healthy survivor effect within this community-based cohort.

**Hispanic group**: While English proficiency was significantly associated with LoI across both NH Black and Hispanic participants, it emerged as a dominant structural barrier among Hispanic older adults. Limited English proficiency exerted both a strong direct association with higher LoI and an indirect association via impaired body function and physical performance. This finding suggests that language barriers can restrict access to community resources essential for maintaining physical independence [59].

### 4.4. Synthesis and Practical Implications

These results underscore the multidimensionality of LoI and demonstrate that risk factors for functional decline are not uniform across populations. Because group-specific pathways—such as language accessibility and household structures—play distinct roles in late-life independence, interventions must move beyond generic aging support. In practice, community and clinical programs should target specific environmental and structural barriers by: (1) Integrating bilingual patient navigators within healthcare settings to mitigate language barriers for Hispanic older adults; and (2) Expanding subsidized, culturally tailored mobility and social participation programs for older adults living alone or navigating complex household dynamics.

### 4.5. Methodological Challenges and Limitations

Several limitations must be considered. First, the cross-sectional design limits any causal inferences; modeled pathways represent statistical associations rather than temporal sequences. Although the path analysis specified directional relationships based on the theoretical ICF framework, the findings represent statistical associations rather than established temporal or causal pathways. Longitudinal research is needed to establish temporal ordering and evaluate these pathways over time. Second, while physical performance was assessed objectively via the SPPB, reliance on self-reported measures for other ICF domains introduces subjective reporting biases. Relatedly, conceptual overlap between the objective SPPB and subjective physical difficulty items on the VES-13 may partially account for the statistical dominance of physical performance in our models. Because this study focuses specifically on mobility-related disability, the SPPB captures objective physical capacity, whereas the VES-13 captures self-reported vulnerability. Furthermore, while complete-case analysis was purposefully utilized to preserve observed multi-ethnic realities, it carries a potential risk of selection bias, as excluded individuals exhibited higher rates of functional vulnerability. Finally, data constraints influenced our contextual and demographic scope. The absence of socioeconomic status (e.g., income, wealth) and structural factors (e.g., healthcare access, discrimination) in Round 13 introduces potential residual confounding that may partly explain observed group variations. Additionally, sample size limitations necessitated excluding smaller racial/ethnic populations (e.g., Asian American, Indigenous adults) and categorizing Hispanic participants as a single group despite internal racial heterogeneity. Future longitudinal research incorporating comprehensive socioeconomic indicators and expanded demographic representation is needed to confirm these group-specific pathways.

## 5. Conclusions

This study demonstrates that Loss of Independence (LoI) is a multidimensional construct influenced by an intersecting array of personal, functional, and environmental factors. Our statistical models demonstrate that while physical performance is a consistent predictor of independence across all groups, the patterns of direct and indirect pathways—such as language and living arrangements—varied across the stratified models. Based on these observed associations, we suggest that policy and practice move beyond generic aging services toward culturally and linguistically responsive interventions. Finally, given the cross-sectional nature of these data, future longitudinal research is needed to confirm the temporal sequences of these group-specific pathways and better inform targeted strategies for preserving independence in later life.

## Figures and Tables

**Table 1:** Characteristics of participants for overall sample and by race/ethnicity (N = 7106).

Characteristics		**Total**		**NH White**		**NH Black**		**Hispanic**		
		n	(%)	n	(%)	n	(%)	n	(%)	*p*-value
**Total**		7106	(100.00)	4393	(82.22)	1392	(8.99)	1321	(8.78)	
**Demographics**										
Age										<0.001
65~74		2460	(52.16)	1367	(50.83)	525	(58.37)	568	(58.22)	
75~85		3150	(37.08)	2003	(37.95)	612	(33.04)	535	(33.09)	
85+		1496	(10.77)	1023	(11.23)	255	(8.59)	218	(8.68)	
Gender										0.043
Men		3037	(45.04)	1928	(45.57)	535	(40.68)	574	(44.57)	
Women		4069	(54.96)	2465	(54.43)	857	(59.32)	747	(55.43)	
		Mean	(SE)	Mean	(SE)	Mean	(SE)	Mean	(SE)	*p*-value
**Loss of Independence (0–10)**		3.87	(0.05)	3.77	(0.06)	4.08	(0.08)	4.6	(0.11)	<0.001
**Health condition**										
Total score	(0–4)	0.92	(0.02)	0.92	(0.02)	0.86	(0.03)	0.96	(0.04)	0.189
**Impaired body function**										
Total score	(0–6)	2.46	(0.02)	2.45	(0.02)	2.48	(0.04)	2.5	(0.05)	0.510
**Physical performance**										
Total score	(0–12)	8.47	(0.09)	8.75	(0.10)	7.12	(0.14)	7.32	(0.17)	<0.001
**Social participation restriction**										
Total score	(0–7)	0.70	(0.02)	0.69	(0.03)	0.82	(0.05)	0.75	(0.05)	0.050
		n	(%)	n	(%)	n	(%)	n	(%)	*p*-value
**Health condition**										
Medical condition	(0–2)	3547	(55.92)	2221	(56.38)	653	(53.67)	673	(53.93)	
	(≥3)	3559	(44.08)	2172	(43.62)	739	(46.33)	648	(46.07)	
Falls in 12 months	(0–1)	5954	(84.50)	3618	(84.11)	1213	(88.11)	1123	(84.38)	
	(≥2)	1128	(15.50)	753	(15.89)	177	(11.89)	198	(15.62)	
Fracture history	Yes	834	(14.62)	531	(15.17)	111	(9.51)	192	(14.68)	
	No	6272	(85.38)	3862	(84.83)	1281	(90.49)	1129	(85.32)	
Hospitalization	Yes	1379	(17.92)	843	(17.69)	279	(18.59)	257	(19.38)	
	No	5173	(82.08)	3538	(82.31)	1112	(81.41)	1063	(80.62)	
**Impaired body function**										
Hearing aid	Yes	1354	(17.90)	1133	(20.41)	89	(4.73)	132	(7.82)	
	No	5750	(82.10)	3258	(79.59)	1303	(95.27)	1189	(92.18)	
Vision aid	Yes	6335	(90.45)	4007	(91.30)	1211	(87.06)	1117	(85.94)	
	No	770	(9.55)	385	(8.70)	181	(12.94)	204	(14.06)	
Chewing/swallowing problems	Yes	756	(9.44)	449	(9.04)	131	(8.71)	176	(13.94)	
	No	6346	(90.56)	3941	(90.96)	1261	(91.29)	1144	(86.06)	
Pain	Yes	4061	(56.79)	2510	(56.85)	834	(59.00)	717	(53.96)	
	No	3044	(43.21)	1882	(43.15)	558	(41.00)	606	(46.04)	
Sleep disturbance	Yes	3351	(44.81)	1851	(42.69)	784	(56.37)	716	(52.87)	
	No	3735	(55.19)	2529	(57.31)	602	(43.63)	604	(47.13)	
Anxiety/depression	Yes	2044	(26.74)	1095	(25.13)	445	(32.43)	504	(35.95)	
	No	5060	(73.26)	3296	(74.87)	947	(67.57)	817	(64.05)	
**Environmental factors**										
Living arrangement										<0.001
Living w/spouse or spouse & others		3443	(56.61)	2335	(59.05)	483	(39.70)	625	(51.11)	
Living alone		2274	(29.16)	1482	(29.39)	494	(34.99)	298	(21.01)	
Living with others only		1389	(14.23)	576	(11.56)	415	(25.31)	398	(27.87)	
Limited English proficiency										<0.001
Well		6394	(95.80)	4380	(99.77)	1383	(99.28)	631	(55.14)	
Not well		712	(4.20)	13	(0.23)	9	(0.72)	690	(44.87)	

Notes: Frequencies (N) are unweighted. Percentages, means, and standard errors are weighted to account for survey design and represent the target population. *p* values reflect a joint significance test comparing all racial/ethnic groups for each variable.

**Table 2:** Standardized Associations of Predictors on Outcome Variables by Racial/Ethnic Groups (N = 7106).

Predictor Variable	Outcome Variable	NH White			NH Black			Hispanic		
		Est.	95% CI		Est.	95% CI		Est.	95% CI	
***Direct effects***										
	LoI									
Female		−0.108 ^‡^	−0.138,	−0.078						
Health condition		0.074 ^‡^	0.040,	0.108	0.202 ^‡^	0.139,	0.264	0.110 ^‡^	0.049,	0.170
Limited English					0.043 ^‡^	0.017	0.069	0.097 ^†^	0.031	0.164
Live w/spouse		0.226 ^‡^	0.190,	0.261	0.180 ^‡^	0.115,	0.245	0.194 ^‡^	0.120,	0.267
Live w/other		0.058 ^‡^	0.030,	0.085	0.139 ^‡^	0.077,	0.202	0.158 ^‡^	0.086,	0.231
Impaired body fx.		0.125 ^‡^	0.097,	0.153	0.072 *	0.011,	0.133	0.137 ^‡^	0.074,	0.200
Phy performance		−0.463 ^‡^	−0.502,	−0.425	−0.388 ^‡^	−0.444,	−0.332	−0.416 ^‡^	−0.473,	−0.359
Soc participation restriction		0.135 ^‡^	0.106,	0.163	0.153 ^‡^	0.109,	0.197	0.104 ^‡^	0.062,	0.146
**Impaired Body Function**										
Female		0.023	−0.014,	0.060	0.064	−0.014,	0.142	0.085 *	0.019,	0.150
Health condition		0.324 ^‡^	0.286,	0.361	0.340 ^‡^	0.287,	0.394	0.378 ^‡^	0.312,	0.443
Limited English		0.024	−0.008,	0.056	0.062 ^†^	0.018,	0.106	−0.053 *	−0.105,	−0.002
Live w/spouse		−0.017	−0.061,	0.026	−0.021	−0.083,	0.041	−0.065	−0.151,	0.020
Live w/other		0.010	−0.033,	0.052	−0.028	−0.098,	0.042	−0.026	−0.113,	0.061
**Phy performance**										
Female		0.043 *	0.005,	0.080	0.063	−0.006,	0.131	−0.031	−0.094,	0.033
Health condition		−0.296 ^‡^	−0.327,	−0.266	−0.295 ^‡^	−0.352,	−0.238	−0.318 ^‡^	−0.389,	−0.248
Limited English		−0.031 *	−0.061,	−0.001	−0.031	−0.107,	0.044	−0.177 ^‡^	−0.254,	−0.100
Live w/spouse		0.140 ^‡^	0.104,	0.175	0.120 ^†^	0.042,	0.199	0.140 ^†^	0.057,	0.224
Live w/other		−0.037	−0.080,	0.007	−0.070 *	−0.133,	−0.008	0.005	−0.068,	0.078
Impaired body function		−0.140 ^‡^	−0.178,	−0.103	−0.075 *	−0.145,	−0.006	−0.085 ^†^	−0.143,	−0.026
**Limited Social participation**										
Female		0.069 ^‡^	0.039,	0.100	0.048	−0.013,	0.110	0.067	−0.009,	0.143
Health condition		0.174 ^‡^	0.136,	0.212	0.177 ^‡^	0.107,	0.247	0.176 ^‡^	0.111,	0.242
Limited English		0.019	−0.022,	0.059	−0.016	−0.067,	0.035	0.022	−0.045,	0.089
Live w/spouse		−0.045	−0.091,	0.002	−0.025	−0.087,	0.037	−0.061	−0.142,	0.019
Live w/other		0.005	−0.040,	0.049	−0.031	−0.097,	0.034	0.040	−0.067,	0.147
Impaired body function		0.197 ^‡^	0.168,	0.227	0.280 ^‡^	0.218,	0.342	0.196 ^‡^	0.146,	0.246
Phy performance		−0.216 ^‡^	−0.254,	−0.178	−0.201 ^‡^	−0.255,	−0.136	−0.170 ^‡^	−0.234,	−0.106
***Indirect effects to LoI***										
**Impaired body function (BF)**										
Female		0.003	−0.002,	0.007	0.005	−0.003,	0.012	0.012 *	0.001,	0.023
Health condition		0.040 ^‡^	0.030,	0.051	0.024 *	0.003,	0.046	0.052 ^‡^	0.026,	0.077
Limited English		0.003	−0.001,	0.007	0.004	0.000,	0.009	−0.007	−0.015,	0.00
Live w/spouse		−0.002	−0.008,	0.003	−0.001	−0.006,	0.003	−0.009	−0.020,	0.003
Live w/other		0.001	−0.004,	0.007	−0.002	−0.007,	0.003	−0.004	−0.016,	0.009
**Phy Performance (PP)**										
Female		−0.020 *	−0.037,	−0.002	−0.024	−0.051,	0.002	0.013	−0.014,	0.039
Health condition		0.137 ^‡^	0.118,	0.157	0.115 ^‡^	0.084,	0.145	0.132 ^‡^	0.091,	0.174
Limited English		0.014 *	0.000,	0.028	0.012	−0.017,	0.042	0.074 ^‡^	0.043,	0.105
Live w/spouse		−0.065 ^‡^	−0.083,	−0.047	−0.047 ^†^	−0.079,	−0.015	−0.058 ^†^	−0.097,	−0.020
Live w/other		0.017	−0.003,	0.037	0.027 *	0.003,	0.052	−0.002	−0.032,	0.028
**Soc participation restriction (SP)**										
Female		0.009 ^‡^	0.005,	0.014	0.007	−0.002,	0.017	0.007	−0.001,	0.015
Health condition		0.023 ^‡^	0.016,	0.031	0.027 ^‡^	0.014,	0.041	0.018 ^‡^	0.008,	0.028
Limited English		0.003	−0.003,	0.008	−0.002	−0.010,	0.006	0.002	−0.005,	0.009
Live w/spouse		−0.006	−0.013,	0.001	−0.004	−0.013,	0.006	−0.006	−0.015,	0.002
Live w/other		0.001	−0.005,	0.007	−0.005	−0.015,	0.005	0.004	−0.007,	0.016
	BF → PP									
Female		0.001	−0.001,	0.004	0.002	−0.001,	0.005	0.003	−0.001,	0.007
Health condition		0.021 ^‡^	0.014,	0.028	0.010 *	0.000,	0.020	0.013 ^†^	0.004,	0.023
Limited English		0.002	0.000,	0.004	0.002	0.000,	0.004	−0.002	−0.004,	0.000
Live w/spouse		−0.001	−0.004,	0.002	−0.001	−0.002,	0.001	−0.002	−0.006,	0.002
Live w/other		0.001	−0.002,	0.003	−0.001	−0.003,	0.001	−0.001	−0.004,	0.003
	BF → SP									
Female		0.001	0.000,	0.002	0.003	0.000,	0.006	0.002 *	0.000,	0.003
Health condition		0.009 ^‡^	0.006,	0.011	0.015 ^‡^	0.009,	0.020	0.008 ^‡^	0.004,	0.012
Limited English		0.001	0.000	0.001	0.003 *	0.001	0.005	−0.001	−0.002	0.000
Live w/spouse		0.000	−0.002,	0.001	−0.001	−0.004,	0.002	−0.001	−0.003,	0.000
Live w/other		0.000	−0.001,	0.001	−0.001	−0.004,	0.002	−0.001	−0.002,	0.001
	PP → SP									
Female		−0.001 *	−0.002,	0.000	−0.002	−0.004,	0.000	0.001	−0.001,	0.002
Health condition		0.009 ^‡^	0.006,	0.011	0.009 ^‡^	0.005,	0.014	0.006 ^‡^	0.003,	0.009
Limited English		0.001	0.000	0.002	0.001	−0.001	0.003	0.003 ^†^	0.001,	0.005
Live w/spouse		−0.004 ^‡^	−0.006,	−0.002	−0.004 *	−0.007,	−0.001	−0.002 *	−0.005,	0.000
Live w/other		0.001	0.000,	0.002	0.002 *	0.000,	0.004	0.000	−0.001,	0.001
	BF → PP → SP									
Female		0.000	0.000,	0.000	0.000	0.000,	0.000	0.000	0.000,	0.000
Health condition		0.001 ^‡^	0.001,	0.002	0.001	0.000,	0.002	0.001 *	0.000,	0.001
Limited English		0.000	0.000,	0.000	0.000	0.000	0.000	0.000	0.000,	0.000
Live w/spouse		0.000	0.000,	0.000	0.000	0.000,	0.000	0.000	0.000,	0.000
Live w/other		0.000	0.000,	0.000	0.000	0.000,	0.000	0.000	0.000,	0.000
***Total effects on LoI***										
Female		−0.115 ^‡^	−0.147,	−0.083	−0.009	−0.035,	0.016	0.037 *	0.007,	0.066
Health condition		0.315 ^‡^	0.286,	0.344	0.402 ^‡^	0.355,	0.450	0.340 ^‡^	0.271,	0.408
Limited English		0.023 *	0.005,	0.040	0.063 ^†^	0.019,	0.106	0.166 ^‡^	0.104,	0.228
Live w/spouse		0.147 ^‡^	0.105,	0.190	0.123 ^‡^	0.055,	0.190	0.114 ^†^	0.036,	0.191
Live w/other		0.078 ^‡^	0.038,	0.119	0.160 ^‡^	0.086,	0.233	0.156 ^‡^	0.071,	0.240
***R*** **^2^**										
LoI		0.382 ^‡^	0.357,	0.408	0.370 ^‡^	0.329,	0.411	0.366 ^‡^	0.311,	0.421
Impaired body function		0.110 ^‡^	0.087,	0.134	0.129 ^‡^	0.088,	0.170	0.168 ^‡^	0.121,	0.215
Phy performance		0.174 ^‡^	0.149,	0.200	0.142 ^‡^	0.105,	0.179	0.210 ^‡^	0.141,	0.279
Soc participation restriction		0.207 ^‡^	0.182,	0.233	0.238 ^‡^	0.191,	0.285	0.209 ^‡^	0.152,	0.266

Note. Limited English = Limited English proficiency; Phy performance = physical performance; Soc = social; LoI = Loss of independence; CI = confidence interval. Est = standardized estimate. * *p* < 0.05 ^†^ *p* < 0.01 ^‡^ *p* < 0.001.

## Data Availability

Publicly available datasets were analyzed in this study. The data supporting these findings can be accessed through the National Health and Aging Trends Study (NHATS) repository at https://www.nhats.org/nhats, accessed on 16 May 2024.

## Supplementary Materials

The following supporting information can be downloaded at: https://www.mdpi.com/article/10.3390/healthcare14172710/s1, Supplementary Table S1. Fit Indices for Selected White, Black and Hispanic Models.
