# Supplementary material for: Loss of Independence Among Racially and Ethnically Diverse Older Adults: An ICF-Based Analysis of NHATS
Source: Healthcare (Basel). 2026 Aug 25;14(17):2710. doi: 10.3390/healthcare14172710 (PMC13565604; doi:10.3390/healthcare14172710)
Supplement: Supplementary file 1 [file healthcare-14-02710-s001.zip › healthcare-4425534-supplementary.pdf]

Supplementary Table S1.

Fit Indices for Selected White, Black and Hispanic Models

	Models	*TRd*[**], *P*	$X^2_{(df)}$, *P*	RMSEA (90% CI)	SRMR	CFI	AIC
White	Model 1[*]	47.1, < .001	$9.936_{(1)}$, .002	.045 (.023, .072)	.005	.997	69298.02
	Model 2		$76.889_{(2)}$, .000	.092 (.075, .111)	.018	.973	69372.64
Black	Model 1*	93.4, < .001	$4.738_{(1)}$, .030	.052 (.013, .102)	.008	.996	22692.98
	Model 2		$76.018_{(2)}$, .000	.163 (.133, .195)	.028	.919	22758.70
Hispanic	Model 1*	8.2, .004	$.036_{(1)}$, .850	.000 (.000, .041)	.001	1.000	21453.67
	Model 2		$9.077_{(2)}$, .011	.052 (.021, .088)	.016	.991	21470.14

Note. [*] Final model in each racial/ethnic group. [**]TRd compares Model 1 to Model 2.
